# AZD2014, a dual mTOR inhibitor, attenuates cardiac hypertrophy in vitro and in vivo

**DOI:** 10.1186/s13036-021-00276-3

**Published:** 2021-10-21

**Authors:** Byung-Hyun Cha, Minjin Jung, Angela S. Kim, Victoria C. Lepak, Brett A. Colson, David A. Bull, Youngwook Won

**Affiliations:** 1 Division of Cardio-Thoracic Surgery, Department of Surgery, University of Arizona College of Medicine, Tucson, AZ 85724 USA; 2 Department of Cellular & Molecular Medicine, University of Arizona College of Medicine, Tucson, AZ 85724 USA

**Keywords:** AZD2014, Cardiomyocyte, Cardiac hypertrophy, mTOR inhibitor

## Abstract

Cardiac hypertrophy is one of the most common genetic heart disorders and considered a risk factor for cardiac morbidity and mortality. The mammalian target of rapamycin (mTOR) pathway plays a key regulatory function in cardiovascular physiology and pathology in hypertrophy. AZD2014 is a small-molecule ATP competitive mTOR inhibitor working on both mTORC1 and mTORC2 complexes. Little is known about the therapeutic effects of AZD2014 in cardiac hypertrophy and its underlying mechanism. Here, AZD2014 is examined in in vitro model of phenylephrine (PE)-induced human cardiomyocyte hypertrophy and a myosin-binding protein-C (*Mybpc3*)-targeted knockout (KO) mouse model of cardiac hypertrophy. Our results demonstrate that cardiomyocytes treated with AZD2014 retain the normal phenotype and AZD2014 attenuates cardiac hypertrophy in the *Mybpc3*-KO mouse model through inhibition of dual mTORC1 and mTORC2, which in turn results in the down-regulation of the Akt/mTOR signaling pathway.

**Supplementary Information:**

The online version contains supplementary material available at 10.1186/s13036-021-00276-3.

## Introduction

Hypertrophic cardiomyopathy (HCM) is a genetic heart disorder with the occurrence rate of 0.2–0.5% [1, 2]. HCM is defined as an increase in cardiac mass, which leads to an increase in the left ventricular mass referred to as left ventricular hypertrophy (LVH) [3]. HCM leads to systolic and diastolic ventricular dysfunction, arrhythmias, sudden cardiac death, and histopathologic changes, such as myocyte disarray and myocardial fibrosis [4, 5]. To date, none of the available pharmacological agents have shown to prevent the process of cardiac hypertrophy and cure the disease [6, 7], with the possible exception of diltiazem [8] for treatment of HCM patients.

The mammalian target of rapamycin (mTOR) pathway appears to be involved in the development of HCM and is considered a therapeutic target for this disease [9–11]. mTOR is an atypical serine/threonine kinase that belongs to the family of the phosphoinositide 3-kinase (PI3K) related kinase and its primary function in a cell is driven by the formation of mTOR complex 1 (mTORC1) and mTOR complex 2 (mTORC2) [12]. The Akt/mTOR pathway contributes significantly to the activation of mTORC1 during the development of cardiac hypertrophy [13], while mTORC2 is involved in the regulation of cell survival, growth, and proliferation in cardiomyocytes [14]. Genetic deletion of mTORC1/2 in a heart impairs the development of cardiac hypertrophy [15]. Preclinical results reveal that a dual mTORC1/2 inhibitor may be superior to a single mTORC1 inhibitor [16, 17]. A new agent capable of blocking the dual mTORC1/2 would be an effective means to prevent or attenuate the progress of cardiac hypertrophy and reduce the proliferation of cardiac progenitor cells or cardiomyocytes in the hypertrophic region.

One of the well-known mTOR inhibitors, rapamycin, a bacterial product and inhibitor of mTORC1, is well-established as an anti-tumor agent or immunosuppressant [18, 19]. In addition to the interest in oncology, many studies have shown that rapamycin diminishes the cardiomyocyte hypertrophy by inhibiting mTORC1 [20]. Recently, several second-generation mTOR inhibitors are under development aimed to overcome the resistance caused by the compensatory up-regulation of mTORC2. One of the dual mTORC1/2 inhibitors, AZD2014, is under clinical stage development in oncology fields [21]. AZD2014 has shown significantly improved inhibitory activity for the mTOR pathway as compared to rapamycin. This is because the inhibition of mTORC2 prevents the feedback activation of Akt signaling, which occurs when only mTORC1 is inhibited [22]. Dual mTORC1/2 inhibitors including AZD2014 have been considered to be more potent agents in cardiac hypertrophy than rapamycin. The benefits of inhibiting the dual mTORC1/2 pathway still remains to be determined in the regulation of cardiac hypertrophy.

Here, we demonstrate that a dual mTORC1/2 inhibitor, AZD2014, modulates both mTORC1 and mTORC2 signaling in cardiomyocytes under the hypertrophic condition. It is investigated whether the inhibition of mTORC1/C2 signaling by AZD2014 is able to control the Akt/mTOR signaling in cardiomyocytes and subsequently attenuate or prevent phenylephrine (PE)-induced human cardiomyocyte hypertrophy in vitro. Finally, we evaluated the anti-hypertrophic activity of AZD2014 in a *Mybpc3*-KO mouse model.

## Materials and methods

### Cell lines and culture conditions

AC16 human adult ventricular cardiomyocyte cells were purchased from the American Type Cell Culture (ATCC). The cells were maintained in Dulbecco’s Modified Eagle’s (DMEM)/F-12 medium (Gibco Invitrogen), supplemented with 12.5% (v/v) fetal bovine serum (Gibco Invitrogen) and 1% (v/v) penicillin/streptomycin (Gibco Invitrogen) at 37 °C in a humidified incubator containing 95% O_2_ and 5% CO_2_. The media was refreshed every 3 days. Cells cultured to approximately 80% confluence were treated with PE (100 μM, Sigma) alone under serum-free conditions, and both rapamycin (100 nM, Sigma), or AZD2014 (range from 10 to 100 nM, dissolved in distilled water, Selleck) under serum conditions with daily treatment.

### Western blot analysis

Cells were washed with Dulbecco’s Phosphate-Buffered Saline (DPBS, Gibco Invitrogen) lysed with 200 μL of radioimmunoprecipitation assay buffer (RIPA buffer), and centrifuged at 13,000 rpm for 20 min. Supernatant was collected in 1.5 mL tubes. Protein concentration was determined using a bicinchoninic acid (BCA) assay kit (Life Technologies). 20 μg of total protein of samples was separated by SDS-polyacrylamide gel electrophoresis (PAGE) and then these proteins were transferred to polyvinylidene fluoride (PVDF) membranes (Millipore). Protein transferred membranes were blocked with 5% (v/v) of skim milk in tris-buffered saline (TBS) containing 0.1% Tween-20 (TBS-T) for 30 min. Membranes were washed three times with TBS-T and incubated with primary antibody in 5% (v/v) skim milk in TBS-T at 4 °C for overnight. After overnight incubation, membranes were washed three times with TBS-T and incubated with secondary antibody in 5% skim milk in TBS-T at RT for 1 h. Immunoreactivity was detected using Clarity™ western ECL substrate (Bio-Rad) and by G:BOX Chemi XX6/XX9 gel doc systems (Syngene, Cambridge, UK). The The total-Akt (t-Akt), phospho-Akt_s473_ (p-Akt_s473_), total-mTOR (t-mTOR), phospho-mTOR_s2448_ (p-mTOR_s2448_), total-S6 (t-S6), phospho-S6_s240 + S244_ (p-S6_s240 + S244_) antibodies were obtained from Abcam. The horseradish peroxidase-conjugated anti-mouse IgG secondary antibody and anti-rabbit IgG were obtained from Abcam. Signals of t-Akt, p-Akt_s473_, t-mTOR, p-mTOR_s2448_, t-S6 and p-S6_s240 + S244_ were quantitatively analyzed using NIH ImageJ software.

### RNA isolation and quantitative real-time polymerase chain reaction (qRT-PCR)

Total RNA was isolated using TRIzol reagent (Invitrogen) according to the manufacturer’s protocol. mRNA was reverse-transcribed into complementary DNA (cDNA) using iScript™ cDNA synthesis kit (Bio-Rad). The quantitative PCR analysis was performed by iTaq Universal SYBR Green Supermix (Bio-Rad) with a CFX96 Touch Real-Time PCR Detection System (Bio-Rad) according to the instructions. Target gene expression was normalized to the glyceraldehyde-3-phosphate dehydrogenase (GAPDH) gene for quantification. The resulting templates were subjected to PCR using the following specific primers: *GAPDH* (sense 5′-ACCCACTCCTCCACCTTTGAC-3′, antisense 5′-TGTTGCTGTAGCCAAATTCGTT-3′); *ANP* (atrial natriuretic peptide, sense 5′-GACAGACTGCAAGAGGCTCC-3′, antisense 5′-GCTGCAGCTTAGATGGGATGA-3′), and *BNP* (B-type naturetic peptide, sense 5′-TCAGCCTCGGACTTGGAAAC-3′, antisense 5′-CTTCCAGACACCTGTGGGAC-3′).

### FACS analysis

Cell surface antigens on cells were evaluated by fluorescence activated cell sorting (FACS) analysis. For FACS, cells were dissociated by 0.05% trypsin/EDTA (Highclone), washed with PBS, fixed with 4% paraformaldehyde, permeabilized with Triton X-100, and blocked with a mixture of bovine serum albumin (BSA). Next, samples were stained with antibodies against Anti-Myh6 Actin (alpha myosin heavy chain actin, Abcam, 1:200) and Anti-ANP antibody (R&D, 1:200) for 30 min or 1 h at 4 °C. Also, Anti-Goat IgG was used for isotype control. Samples were subsequently stained with fluorescently labeled secondary antibodies (Alexa Fluor-488 conjugated goat anti-mouse (Abcam, 1:400) and Alexa Fluor-647 conjugated donkey anti-goat (Abcam, 1:400)) for 30 min at 4 °C. Corresponding mouse/rabbit isotype antibodies were used as controls. Cell immunotypes were determined by BD FACS CantoII (BD Biosciences) and the percentage of expressed cell surface antigen was calculated for 10,000 gated-cell events.

### Immunostaining

Samples were fixed with 4% paraformaldehyde, permeabilized with Triton X-100, and blocked with a mixture of BSA. Samples were incubated with primary antibodies (Anti-Myh6 Actin (Abcam, 1:200) and Anti-ANP antibody (R&D, 1:200) overnight at 4 °C and washed. Samples were subsequently stained with fluorescently labeled secondary antibodies (Alexa Fluor-488 conjugated goat anti-mouse (Abcam, 1:400), Alexa Fluor-647 conjugated donkey anti-goat (Abcam, 1:400)) for 30 min at at RT and 4′,6-diamidino- 2-phenylindole dihydrochloride (DAPI) as a nuclear counterstain for 5 min at R.T. Samples were washed and mounted for imaging on a fluorescence microscope (Leica DMI-6000, Leica). The relative surface area of coverage for stains was quantified with ImageJ software (NIH).

### Administration of AZD2014

Myosin-binding protein C (*Mybpc3*)-targeted knockout (KO) mice recapitulate typical aspects of human hypertrophic cardiomyopathy. *Mybpc3*-targeted ablation in the KO mice is sufficient to trigger profound cardiac hypertrophy, impaired systolic and diastolic function, and depressed myocyte contractile properties [23]. All experiments were approved by the University of Arizona Institutional Animal Care and Use Committee and followed the U.S. National Institutes of Health Using Animals in Intramural Research guidelines for animal use. Seven-week-old *Mybpc3* KO mice (male) were used to examine the effect of AZD2014. The mice were given AZD2014 at doses of 2.5 mg or 10 mg/kg on IP injection twice a week for 12 weeks.

### Echocardiography

Survival was monitored and echocardiography was performed at 12 weeks, at the end of the course of treatment. All imaging studies were performed using the VisualSonicsVevo® 770 imaging system (Scanhead: RMV707B, 15–45 MHz). Anesthesia was induced for 1 minute in an induction chamber (3% isoflurane mixed with 97% O_2_ at a flow rate of 1 L/min). After placing the mouse in a supine position atop a pad with embedded ECG electrodes, anesthesia was maintained via inhalation of 1.5–2% isoflurane and 98–98.5% O_2_ at a flow rate of 1 L/min using a nose mask. The ECG signal was monitored throughout the procedure. After immobilizing the mouse on the echocardiography stage with tape, chest hair was removed with hair removal cream and a layer of preheated ultrasound gel was applied to the chest. Body temperature was monitored throughout the whole procedure by an inserted rectal probe and maintained within a narrow range (37.0 °C ± 1.5 °C) via the heated platform and a heat lamp. Standard measurements of were performed in systole and diastole in parasternal long-axis projection and the functional parameters were calculated from M-mode measurements.

### Histology

At the endpoint of each experiment, mice were anaesthetized with 4% isoflurane and subsequently euthanized by cervical dislocation. The heart was harvested, washed, and fixed for histological analyses. Cross-sections of the hearts made along the transverse midventricular tissue were stained for hematoxylin and eosin (H&E).

### Statistical analysis

Data are expressed as means ± standard deviation (SD) from at least three independent experiments and were analyzed using one-way ANOVA and Tukey’s post-hoc test. In all cases, *p* < 0.05 was considered to be statistically significant.

## Results

### Effect of AZD2014 on phenylephrine (PE)-induced hypertrophic cardiomyocytes

**Fig. 1:**
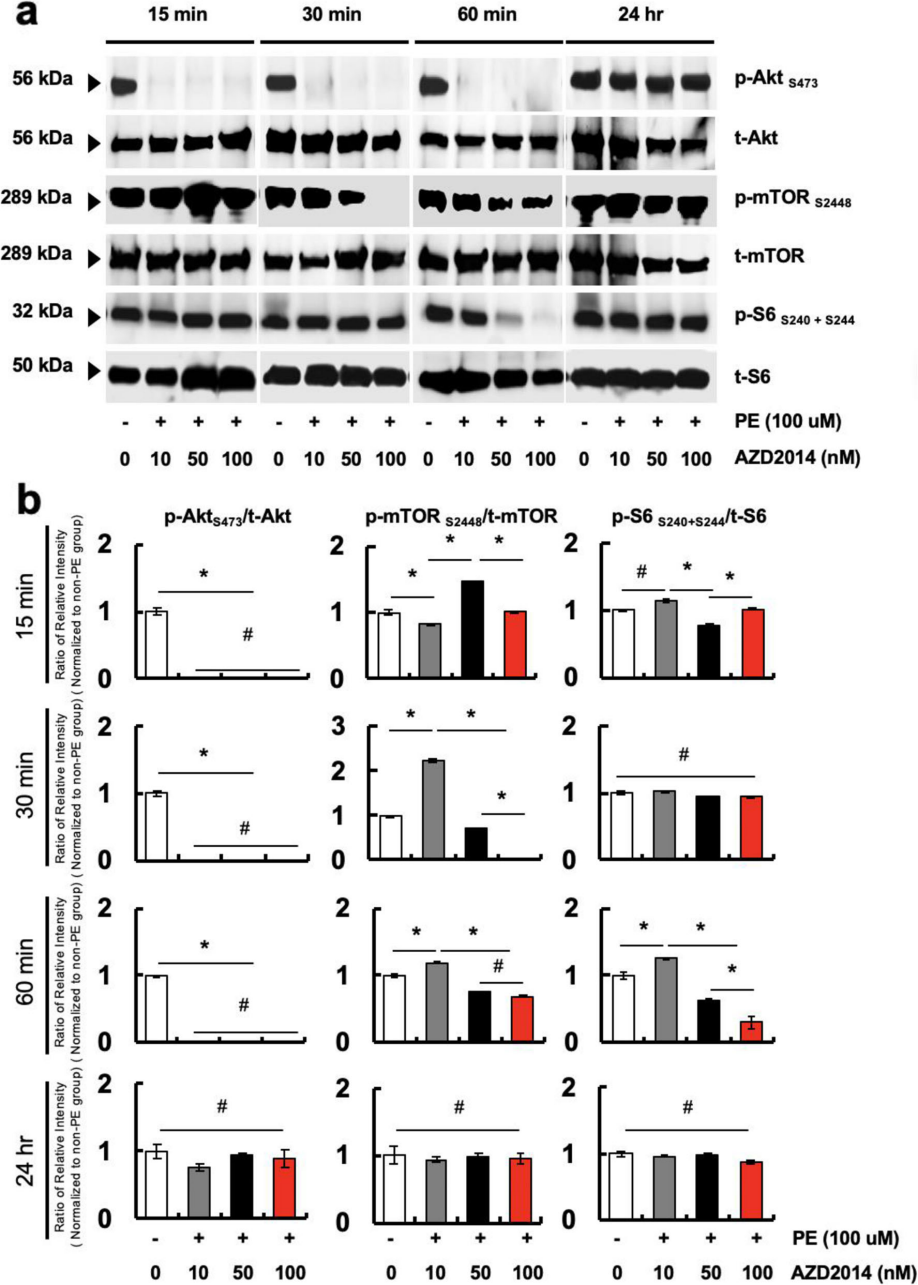
Time course of changes in myocardial mTOR signaling in phenylephrine (PE)-induced cardiac hypertrophy due to AZD2014 treatment. **a** Effects of AZD2014 on Akt, mTOR and S6 phosphorylation from PE-induced cardiac hypertrophy evaluated by western blot. **b** Quantitative densitometry of phosphorylated/total protein expression from western blots by ImageJ. #, *p* > 0.05; *, *p* < 0.05. *n* = 3

Expression of proteins related to myocardial mTORC1/2 signaling pathways was examined in hypertrophic cardiomyocytes. Treatment of cardiomyocytes with PE mimics the features of pathological hypertrophy, such as increased cytosolic and nucleus area in vitro [24]. Cells were pre-incubated in serum-free medium for 24 h prior to additional 24 h incubation with or without PE (100 μM). After the hypertrophy induction by PE, cardiomyocytes were treated with AZD2014 at various concentrations ranging from 10 nM to 100 nM from 15 min up to 24 h (Fig. 1). The band images and relative intensities of the phosphorylated/total protein ratio showed that the mTOR_S2448_ (mTORC1) and Akt_S473_ (mTORC2) signaling in cardiomyocytes were most inhibited by AZD2014 at the condition of 30 min treatment at 100 nM (Fig. 1a-b). Reduction of phosphorylation of p-S6_S240 + S244_ (mTORC1) occurred later as a consequence of the mTORC1 inhibition (an additional 30 min). The inhibitory effects of AZD2014 faded out in a time-dependent manner up to 24 h of observation.

### AZD2014 attenuates PE-induced cardiomyocyte hypertrophy

**Fig. 2:**
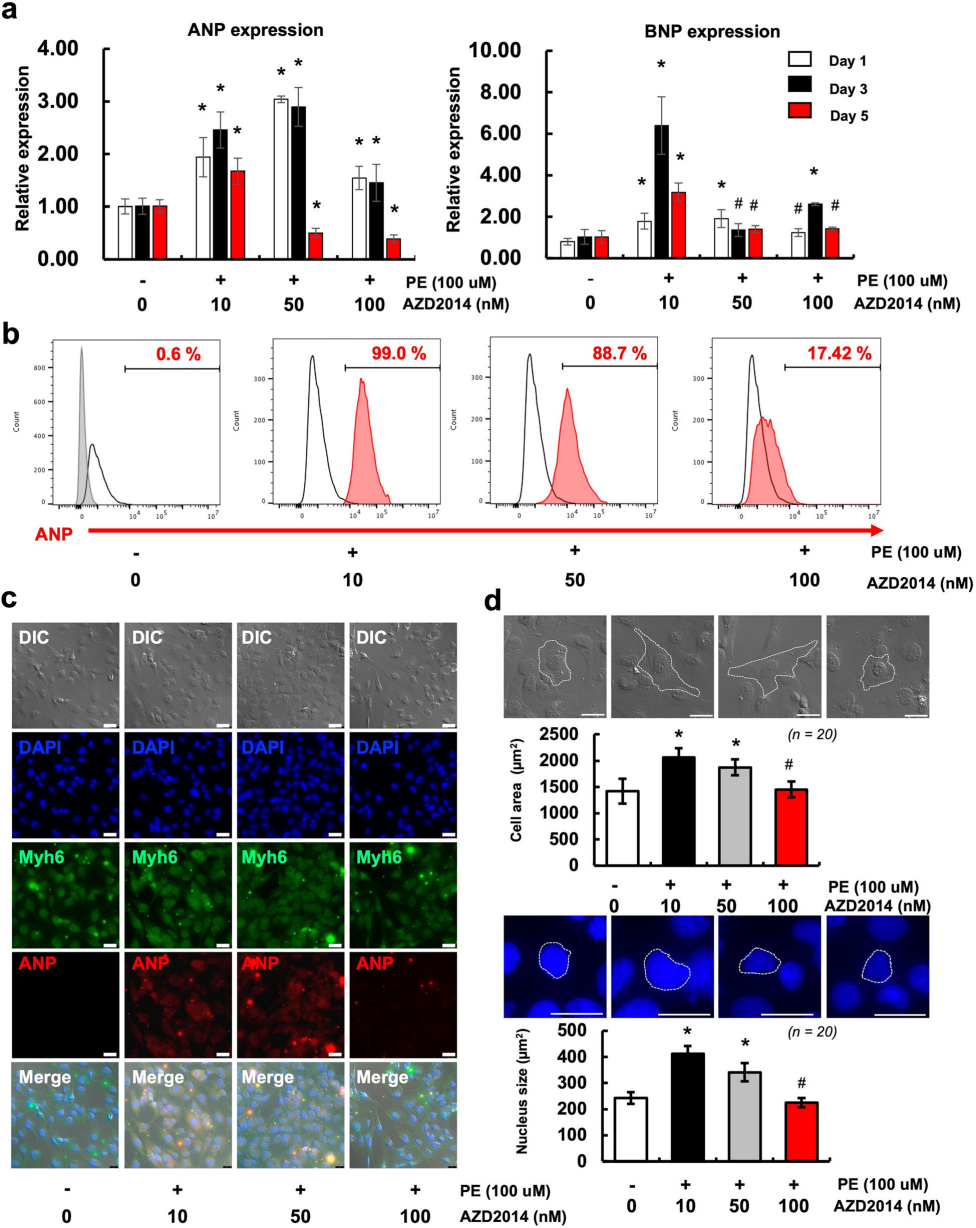
Effects of AZD2014 on phenylephrine (PE)-induced cardiac hypertrophy. **a** The mRNA expression levels of cardiac hypertrophy marker *ANP* and *BNP* in cardiomyocytes treated as indicated were determined by qRT-PCR. #, *p* > 0.05; *, *p* < 0.05 vs. non-PE treated group. *n* = 3. **b** The protein expression levels of ANP in cardiomyocytes treated as indicated were determined by FACS (The gray shaded peak is the isotype control). **c** Representative fluorescence microscopy images of immunocytochemistry for cardiomyocyte marker Myh6 and ANP in cardiomyocytes treated as indicated. Scale bar = 25 μm. **d** Cell area and nucleus size in cardiomyocytes treated as indicated were determined by microscopy and Image J. Graphic representation of cell area and nuclear size from 20 randomly selected measured cells. #, *p* > 0.05; *, *p* < 0.05 vs. non-PE treated group. Scale bar = 25 μm

To investigate the effect of AZD2014 on cardiomyocyte hypertrophy, cardiomyocytes were treated with or without PE (100 μM) followed by AZD2014 treatments (10, 50, or 100 nM) for 1, 3, or 5 days (Fig. 2). Initially, the exposure to PE markedly upregulated cardiomyocyte hypertrophy markers, atrial natriuretic peptide (*ANP*) and B-type natriuretic peptide (*BNP*), but this effect was highly diminished by the treatment with AZD2014 in a dose-dependent manner (Fig. 2a). The expression of the cardiomyocyte hypertrophy markers in all groups decreased during the 5-day treatment period. Especially, at day 5, the expressions of *ANP* and *BNP* in both 50 and 100 nM AZD2014 groups were significantly decreased. As seen in Fig. 2b, the protein expression of ANP drastically decreased in the 100 nM AZD2014-treated group (17.42%) as compared to the other 10 and 50 nM AZD2014-treated groups (99.0 and 88.7%). Consistent with these findings, immunocytochemistry (ICC) results (Fig. 2c) also demonstrate that the protein expression of ANP significantly decreased in the 100 nM AZD2014-treated group as compared to others, while retaining the level of Myh6, which is a marker of fully-differentiated cardiomyocytes. In addition, cardiac hypertrophy is accompanied by an increase in cell area and nuclear enlargement with an increase in cellular DNA content [25]. We measured the cell area and the nuclear size on cardiomyocytes upon treatment of AZD2014. As seen in Fig. 2d, PE-induced hypertrophic cardiomyocytes have morphological characteristics found in cardiomyocytes undergoing hypertrophy, which include widespread cytosolic area (central 2 DIC images of top panels) and an enlarged nucleus (central 2 DAPI images of top panels). Treatment of AZD2014 at 10 or 50 nM was ineffective to inhibit this process. However, interestingly, the cell area and nucleus size were markedly reduced in 100 nM AZD2014-treated group. These results indicate that treatment of AZD2014 at 100 nM is sufficient to delay the onset of cardiac hypertrophy and retain a normal phenotype in cardiomyocytes.

### The effect of AZD2014 and rapamycin was similar in progression of cardiomyocyte hypertrophy

**Fig. 3:**
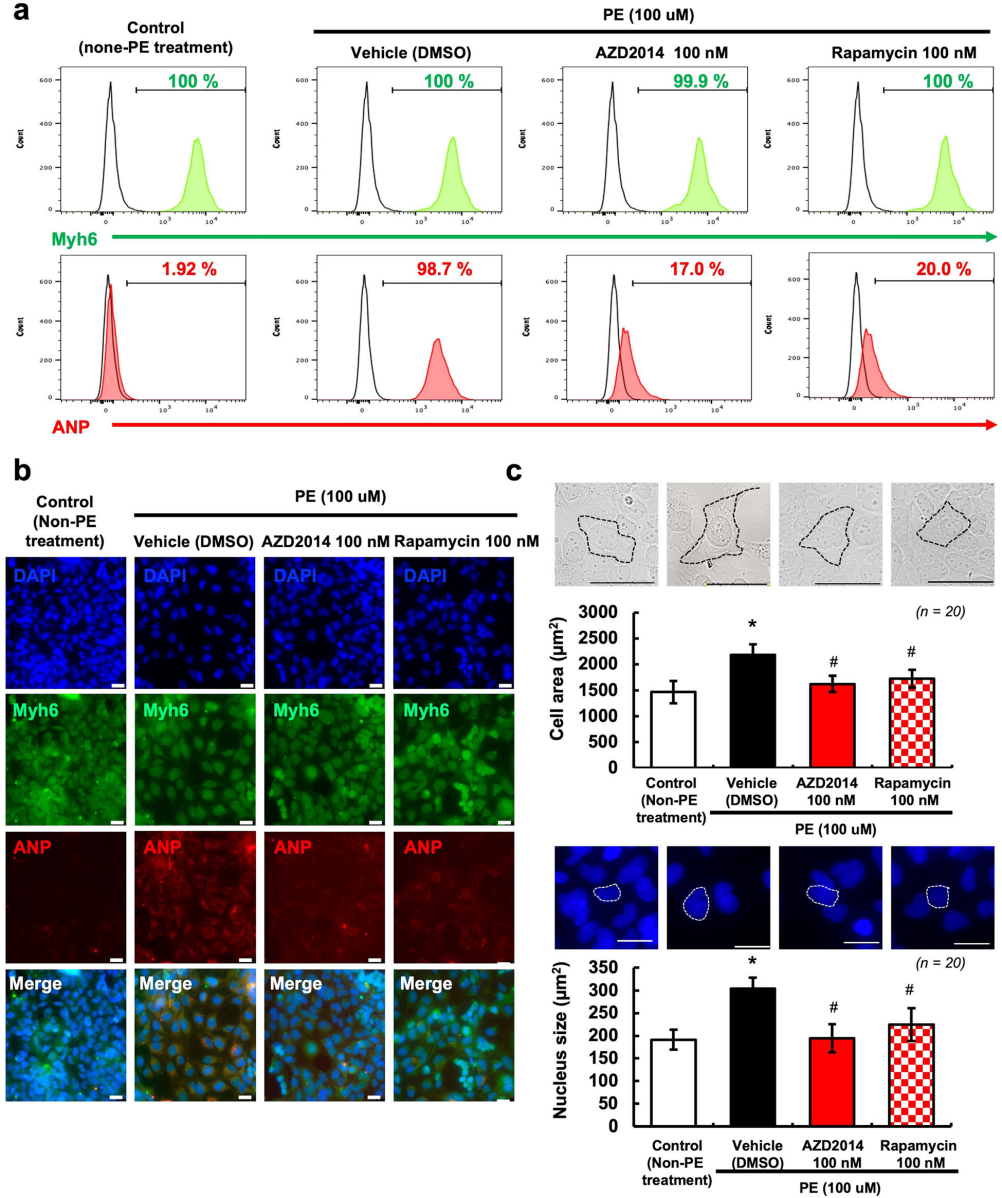
Comparison of AZD2014 (100 nM) and Rapamycin (100 nM) on phenylephrine (PE)-induced cardiac hypertrophy. **a** The protein expression levels of Myh6 and ANP in cardiomyocytes treated as indicated were determined by FACS. **b** Representative fluorescence microscopy images of immunocytochemistry for Myh6 and ANP of cardiomyocytes treated as indicated. Scale bar = 25 μm. **c** Cell area and nucleus size in cardiomyocytes treated as indicated were determined by fluorescence microscopy and Image J. Graphic representation of cell area and nuclear size from 20 randomly selected measured cells. #; *p* > 0.05, *; *p* < 0.05 vs. Control (non-PE treatment). Scale bar = 25 μm

To compare the effects of AZD2014 and rapamycin (as well-known allosteric inhibitors of mTORC1) in PE-induced cardiomyocyte hypertrophy, cardiomyocytes were treated with or without PE (100 μM) and then cultured with media containing AZD2014 (10, 50, or 100 nM) or rapamycin (100 nM) for 5 days (Fig. 3). As seen in Fig. 3a, markers of differentiated cardiomyocytes, Myh6 were well-expressed in all groups. However, the protein expression of ANP were drastically decreased in both 100 nM AZD2014 (17.0%) and 100 nM rapamycin (20.0%) -treated groups as compared to the vehicle group (98.7%). Consistent with these findings, the protein expression of ANP was significantly decreased in the 100 nM AZD2014 and the 100 nM rapamycin -treated groups as compared to the vehicle group (Fig. 3b). We also measured the cell area and the nuclear size of cardiomyocytes after treating with AZD2014 or rapamycin. As seen in Fig. 3c, the cell area and the nucleus size were remarkedly increased in PE-induced cardiomyocytes. However, the cell area and the nucleus size were statistically significant reductions in both AZD2014 and rapamycin-treated groups. These results indicated that both 100 nM AZD2014- and rapamycin-treated cardiomyocytes are sufficient to restore a normal phenotype of cardiomyocytes.

### mTOR inhibition by AZD2014 protects against cardiac hypertrophy

**Fig. 4:**
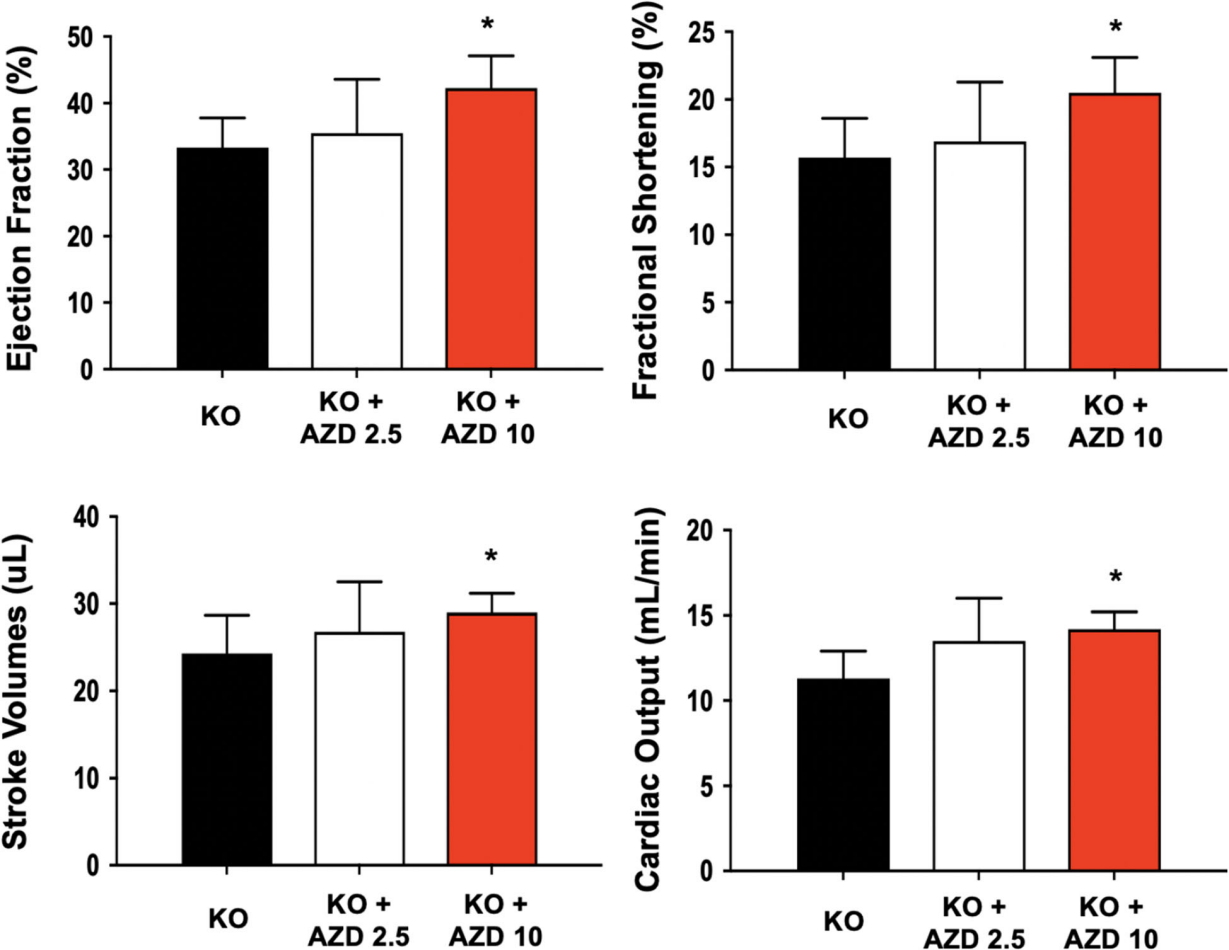
In vivo cardiac functional improvement of *Mybpc3*-KO mouse model via *i.p.* administration of 2.5 mg and 10 mg AZD2014. Echocardiography-based quantification of cardiac functional improvement-related parameters in *Mybpc3*-KO mice from different treatment groups. *n* = 5, animal per group, mean ± S.D., *; *p* < 0.05 vs. KO (by one-way ANOVA)

The efficacy of AZD2014 was examined in *Mybpc3*-KO mice. The effects of AZD2014 on the development of cardiac hypertrophy was investigated in a dose-dependent manner of AZD2014. Twelve weeks after the treatment, the effect of AZD2014 on cardiac function was determined by echocardiography (Fig. 4). Treatment with AZD2014 at 10 mg/kg significantly improved cardiac function of the mice as compared to the sham-treated mice, as assessed by ejection fraction, fractional shortening, stroke volume, and cardiac output. We also examined the mRNA expression of *ANP* and *BNP* in the hearts at the end of the treatment (Supplemental Fig. S1) because cardiac hypertrophy in response to pressure overload is associated with reactivation of “fetal” genes, such include *ANP* and *BNP* [26]. There was a significant decrease in the mRNA expression of *ANP* and *BNP* in the hearts treated with 10 mg/kg of AZD2014 as compared to the sham-treated group. Hearts were excised and tissues were processed for H&E. As shown in Supplemental Fig. S2, the representative H&E stained sections provide histological evidence for the effects of AZD2014 on the prevention of cardiac hypertrophy. These results demonstrate that AZD2014 has the potential to attenuate the development of cardiac hypertrophy in a mouse model of human HCM.

## Discussion

It is well understood that mTOR plays a key role in regulating cell growth, proliferation, and apoptosis/necrosis [27, 28]. In addition, mTOR functions as an important regulator to inhibit mTORC1 during the progression of cardiac hypertrophy [20]. In this study, we examined the effect of AZD2014, a dual mTORC1/2 inhibitor, on PE–induced hypertrophic cardiomyocytes and a *Mybpc3*-KO mouse model of HCM. We found that AZD2014 effectively attenuates PE-induced cardiomyocyte hypertrophy, as evidenced by the reduction in the cardiomyocyte cell/nucleus area and cardiac hypertrophic protein/mRNA markers, which are predicted to be consequences of mTORC1/2 inhibition.

Although the two complexes of mTOR are functionally interconnected, most studies are focused on the role of mTORC1 in the regulation of intracellular signaling [29, 30]. Especially, the recent studies have documented that mTOR inhibition by rapamycin reduced myocardial infarct size, which is evidence to evaluate cardiac hypertrophy, with inhibiting cardiomyocyte apoptosis and preserving cardiac function in aortic-banded mice following myocardial ischemia reperfusion (MI/R) [31]. However, little is known about the role of mTORC2 in the mTOR signaling pathway. Our results of mTOR signaling in human cardiomyocyte cell line (AC16) indicated that p-Akt_ser473_ (mTORC2), mTOR_S2448_ (mTORC1), and p-S6_S240 + S244_ (mTORC1) are rapidly and substantially inhibited upon the treatment of AZD2014 (Fig. 1). We have shown that PE-induced human cardiomyocytes treated with AZD2014 reduced the expression of hypertrophy marker genes, *ANP* and *BNP*, resulting in the decelerated progression of hypertrophy (Fig. 2). It was initially hypothesized that dual inhibition of mTORC1/2 is more effective to block the mTOR pathway in cardiomyocytes regardless of PE-treatment than single inhibition of either mTORC1 or mTORC2. However, in in vitro culture condition on AC16, we observed no significantly difference in the cardiac hypertrophy-related gene expression upon treatment with either AZD2014 or rapamycin (Supplemental Fig. S3). Nevertheless, in terms of the regression of cardiac hypertrophy, AZD2014 exhibited more attractive results attributed from the inactivation of dual mTORC1/2 complexes. These are the first observations of inhibition of Akt/mTOR signaling in human cardiomyocytes by blocking the dual mTOR pathways.

We hypothesized that AZD2014 would be superior for amelioration of HCM pathological remodeling via synergistic effects resulting from the dual inhibition of mTORC1 and mTORC2 over the single mTOR inhibitor. In vivo, the *Mybpc3*-KO mouse model given AZD2014 to dually inhibit mTORC1/2, revealed that mTOR is involved in the regulation of cardiac hypertrophy and in the control of vital cellular processes necessary for maintenance of cardiac function. As a dual mTOR kinase inhibitor, AZD2014 demonstrates preventive effects against PE-induced cardiac hypertrophy in vitro and *Mybpc3*-related HCM phenotypes in vivo. However, the advantages of dual mTOR inhibition over single mTOR inhibition should be further studied. AZD2014 appears to be a potentially highly therapeutic candidate for treating human diseases associated with pathological cardiac hypertrophy.

## Supplementary Information

**Additional file 1: Fig. S1.** mRNA expression of atrial natriuretic peptide (*ANP*) and B-type natriuretic peptide (*BNP*) from *Mybpc3*-KO mouse model following AZD2014 intraperitoneal (i.p.) administration. The mRNA expression levels of cardiac hypertrophy markers *ANP* and *BNP* in hearts were determined by qRT-PCR. #, *p* > 0.05; *, *p* < 0.05. *n* = 3. **Fig. S2.** Representative micrographs of H&E from *Mybpc3*-KO mouse model via *i.p.* administration of 2.5 mg and 10 mg AZD2014 (Red indicates cytoplasm; Blue indicates nucleus). **Fig. S3.** The protein expression of *Myh6* and *ANP* on phenylephrine (PE)-induced cardiomyocyte by treatment of AZD2014 and rapamycin. Cardiomyocytes were treated with or without PE (100 μM) and cultured with AZD2014 and rapamycin in a dose-dependent manner for 5 days. The protein expression of *Myh6* and *ANP* of AZD2014 or rapamycin-treated cardiomyocytes on post-PE-induced hypertrophy were determined by FACS. #, *p* > 0.05. *n* = 3.

## Abbreviations

mTOR: Mammalian target of rapamycin
PE: Phenylephrine
Mybpc3: Myosin-binding protein-C
KO: Knockout
HCM: Hypertrophic cardiomyopathy
LVH: Left ventricular hypertrophy
PI3K: Phosphoinositide 3-kinase
mTORC1: mTOR complex 1
mTORC2: mTOR complex 2
t-Akt: total-Akt
p-Akt_s473_: phospho-Akt_s473_
t-mTOR: total-mTOR
p-mTOR_s2448_: phospho-mTOR_s2448_
t-S6: total-S6
p-S6_s240 + S244_: phospho-S6_s240 + S244_
Myh6: Alpha myosin heavy chain gene
ANP: Atrial natriuretic peptide
BNP: B-type naturetic peptide
GAPDH: Glyceraldehyde-3-phosphate dehydrogenase
