# Supplementary material for: AZD2014, a dual mTOR inhibitor, attenuates cardiac hypertrophy in vitro and in vivo
Source: J Biol Eng. 2021 Oct 21;15:24. doi: 10.1186/s13036-021-00276-3 (PMC8529824; doi:10.1186/s13036-021-00276-3)
Supplement: Supplementary file 1 — Additional file 1: Fig. S1. mRNA expression of atrial natriuretic peptide (ANP) and B-type natriuretic peptide (BNP) from Mybpc3-KO mouse model following AZD2014 intraperitoneal (i.p.) administration. The mRNA expression levels of cardiac hypertrophy markers ANP and BNP in hearts were determined by qRT-PCR. #, p > 0.05; *, p < 0.05. n = 3. Fig. S2. Representative micrographs of H&E from Mybpc3-KO mouse model via i.p. administration of 2.5 mg and 10 mg AZD2014 (Red indicates cytoplasm; Blue indicates nucleus). Fig. S3. The protein expression of Myh6 and ANP on phenylephrine (PE)-induced cardiomyocyte by treatment of AZD2014 and rapamycin. Cardiomyocytes were treated with or without PE (100 μM) and cultured with AZD2014 and rapamycin in a dose-dependent manner for 5 days. The protein expression of Myh6 and ANP of AZD2014 or rapamycin-treated cardiomyocytes on post-PE-induced hypertrophy were determined by FACS. #, p > 0.05. n = 3. [file 13036_2021_276_MOESM1_ESM.docx]

**Supplementary Information**

AZD2014, a dual mTOR inhibitor, attenuates cardiac hypertrophy *in vitro* and *in vivo*

Byung-Hyun Cha ^a^, Minjin Jung ^a^, Angela S. Kim ^a^, Victoria C. Lepak ^b^, Brett A. Colson ^b^, David A. Bull ^a^, Youngwook Won ^a^*

^a^ Division of Cardio-Thoracic Surgery, Department of Surgery, University of Arizona College of Medicine, Tucson, AZ, 85724, USA.

^b^ Department of Cellular & Molecular Medicine, University of Arizona College of Medicine, Tucson, AZ, 85724, USA

*Correspondence and requests for materials should be addressed to Dr. Y.Won. (email: youngwookwon@email.arizona.edu)

**This Supplemental file includes:**

**Figure S1, Figure S2, and Figure S3**


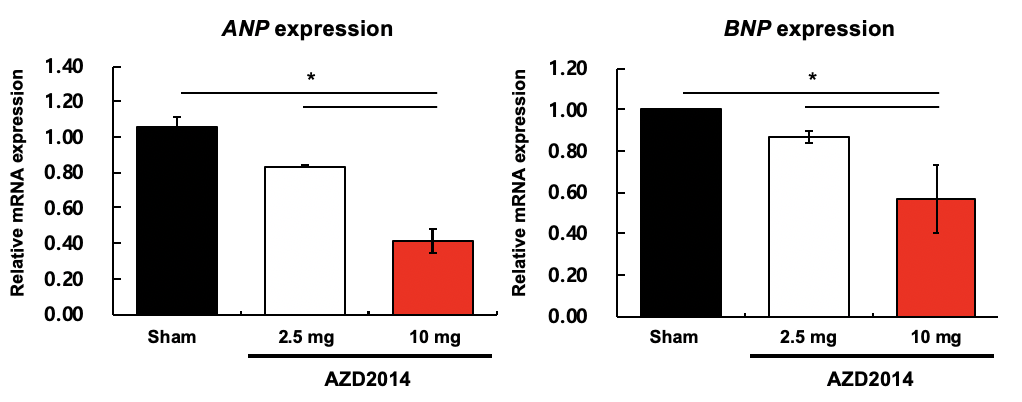


**Fig. S1.** mRNA expression of atrial natriuretic peptide (*ANP*) and B-type natriuretic peptide (*BNP*) from *Mybpc3*-KO mouse model following AZD2014 intraperitoneal (i.p.) administration. The mRNA expression levels of cardiac hypertrophy markers *ANP* and *BNP* in hearts were determined by qRT-PCR. #, *p* > 0.05; *, *p* < 0.05. *n* = 3.


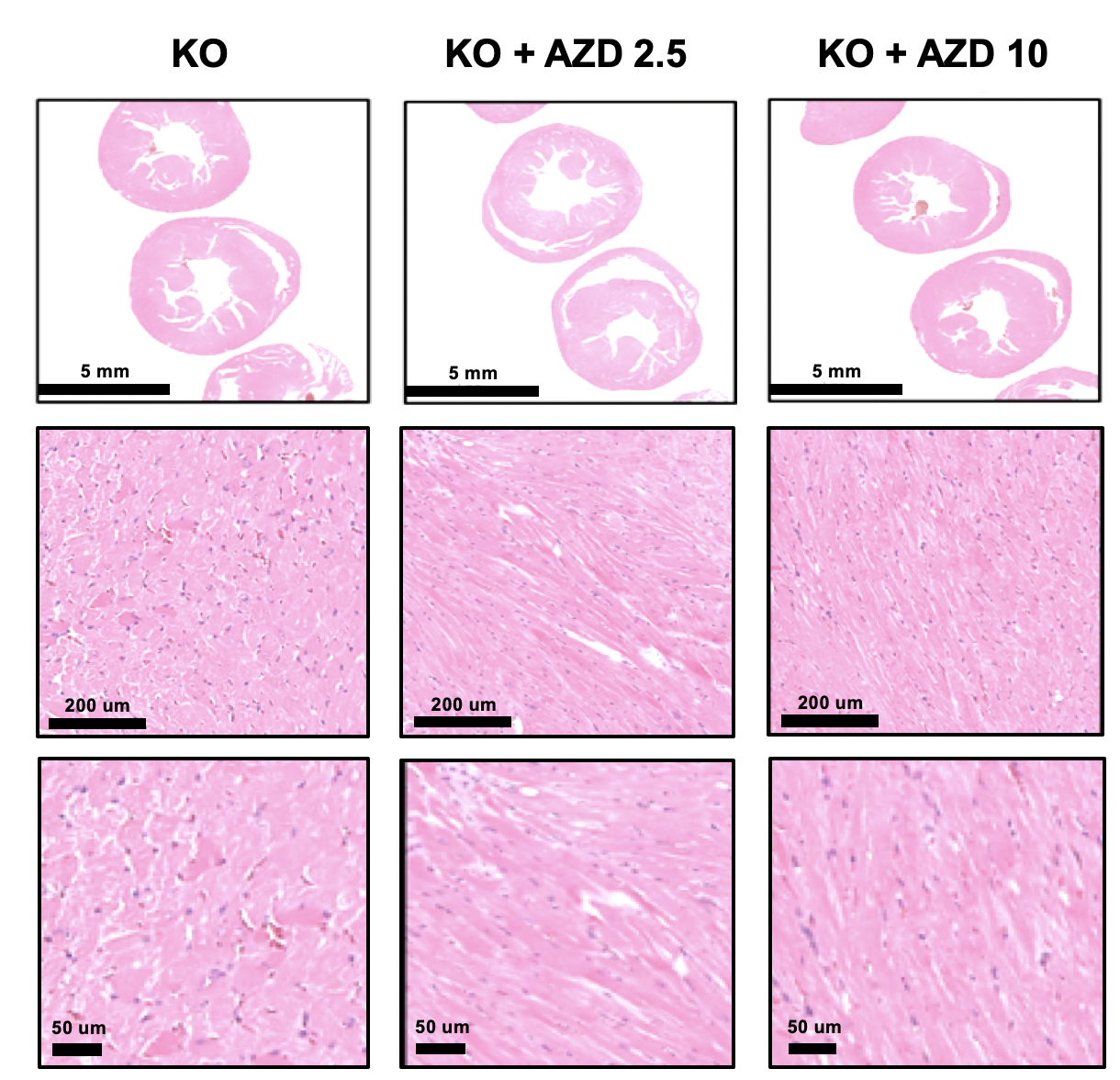


**Fig. S2.** Representative micrographs of H&E from *Mybpc3*-KO mouse model *via* *i.p.* administration of 2.5 mg and 10 mg AZD2014 (Red indicates cytoplasm; Blue indicates nucleus).


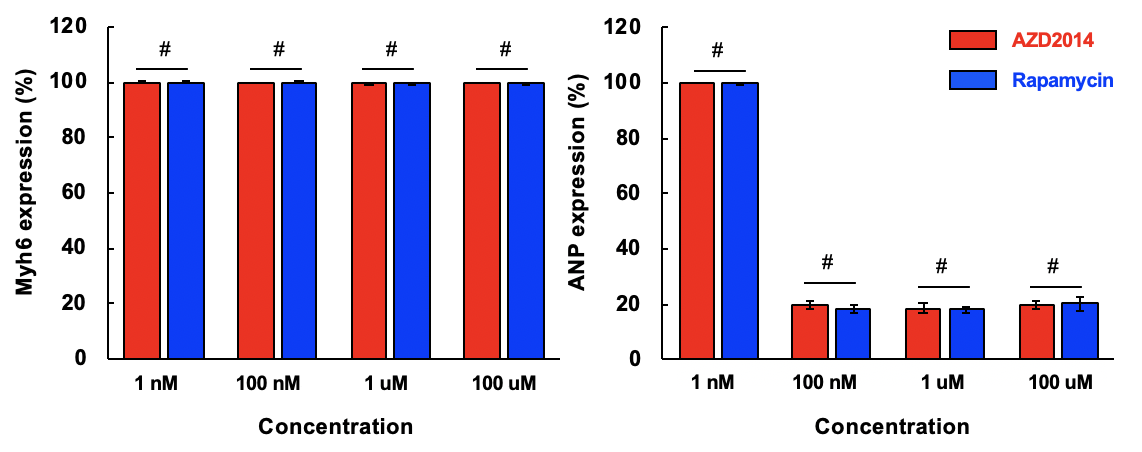


**Fig. S3.** The protein expression of *Myh6* and *ANP* on phenylephrine (PE)-induced cardiomyocyte by treatment of AZD2014 and rapamycin. Cardiomyocytes were treated with or without PE (100 μM) and cultured with AZD2014 and rapamycin in a dose-dependent manner for 5 days. The protein expression of *Myh6* and *ANP* of AZD2014 or rapamycin-treated cardiomyocytes on post-PE-induced hypertrophy were determined by FACS. #, *p* > 0.05. *n* = 3.
